# MIG-6 Is Critical for Progesterone Responsiveness in Human Complex Atypical Hyperplasia and Early-Stage Endometrial Cancer

**DOI:** 10.3390/ijms232314596

**Published:** 2022-11-23

**Authors:** Olivia Jeong, Russell R. Broaddus, Bruce A. Lessey, John I. Risinger, Mark I. Hunter, Tae Hoon Kim

**Affiliations:** 1 Department of Obstetrics, Gynecology & Reproductive Biology, College of Human Medicine, Michigan State University, Grand Rapids, MI 49503, USA; 2 Public Health Sciences Program, University of Michigan School of Public Health, Ann Arbor, MI 48109, USA; 3 Department of Pathology and Laboratory Medicine, University of North Carolina School of Medicine, Chapel Hill, NC 27599, USA; 4 Department of Obstetrics and Gynecology, Atrium Health Wake Forest Baptist, Winston-Salem, NC 27157, USA; 5 Department of Obstetrics, Gynecology and Women’s Health, University of Missouri, Columbia, MO 65211, USA

**Keywords:** progesterone receptor, MIG-6, progesterone resistance, fertility-sparing treatment, endometrial hyperplasia, endometrial cancer

## Abstract

Women with complex atypical hyperplasia (CAH) or early-stage endometrioid endometrial cancer (EEC) are candidates for fertility preservation. The most common approach is progesterone (P4) therapy and deferral of hysterectomy until after completion of childbearing. However, P4 therapy response rates vary, and molecular mechanisms behind P4 resistance are poorly understood. One potential molecular cause of P4 resistance is a loss or attenuation of PGR expression. Mitogen-inducible gene 6 (MIG-6) is critical for P4 responsiveness. MIG-6 protein expression in the endometrial epithelial and stromal cells from women with CAH and EEC was significantly lower compared to women without CAH or EEC. The P4-responsive women (10/15) exhibited an increase of MIG-6 expression in epithelial and stromal cells compared to P4-resistant women (5/15). In addition, immunohistochemical analysis for PGR results showed that stromal PGR levels are significantly higher in P4-responsive women compared to P4-resistant women, whereas epithelial PGR expression was not different. A reverse correlation of MIG-6 and pAKT levels was observed in early-stage EEC patients. Studies strongly suggest that loss of MIG-6 and PGR and activation of pAKT lead to P4 resistance in CAH and EEC. These results will help to elucidate the molecular mechanism leading to P4 resistance in CAH and EEC.

## 1. Introduction

Endometrial cancer (EC) is the most frequent malignancy of the female genital tract in the U.S. [1]. According to the American Cancer Society, there will be ~66,000 new EC cases in the U.S. in 2022 [2], with an annual mortality close to 12,550 [1]. EC is expected to increase due to rising incidence of obesity and type 2 diabetes, which are well-known risk factors for EC in women [3,4,5]. Most women with EC can be cured by hysterectomy, the surgical removal of the uterus [6,7,8]. However, hysterectomy is not an option for all women with EC, including extremely obese women with related cardiovascular disease, women with organ failure due to diabetes, women with ventilation difficulties, and elderly women exposed to a high surgical risk [9,10]. In addition, 20–30% of the young women with EC might be eligible for a fertility-sparing approach [11,12,13]. Developing nonsurgical treatments to cure EC without sacrificing fertility remains an essential goal in EC medicine.

Poor understanding of the mechanism of progesterone (P4) resistance in endometrioid endometrial cancer (EEC) is a major barrier to developing nonsurgical EEC treatments that preserve fertility. EEC, the most common type of endometrial cancer (EC) (80–85%), is associated with or preceded by abnormal multiplication of endometrial epithelial cells, known as complex atypical hyperplasia (CAH) [14,15,16]. CAH is a common type of endometrial hyperplasia that becomes EEC in up to 30% of cases if not treated [14,16,17,18]. CAH is characterized by an increased endometrial gland-to-stroma ratio and endometrial proliferation [19]. P4 signaling disruption unleashes unopposed estrogen (E2) stimulation, which causes CAH to develop into EEC [20,21]. P4 is widely used to treat various gynecological conditions [22] due to its clear antiproliferative effects on E2-mediated endometrial proliferation [23]. P4 can be classified as natural (the endogenous progesterone) or synthetic (progestins) [24]. Current conservative treatment methods mainly involve P4 therapy by oral progestin or by an intrauterine device (IUD) [25,26]. P4, the gold-standard of nonsurgical treatment, is often an effective EEC treatment [27,28,29]: A meta-analysis of 45 studies including women with grade I EEC or CAH who received P4 therapy found durable, complete responses in 53% [30,31,32]. However, molecular mechanisms behind de novo or acquired P4 resistance are poorly understood. To increase P4 therapy success rates and to decrease the risks of fertility-preserving approaches, its essential to reveal the mechanisms underlying P4 resistance in EEC. A closely related barrier to progress is the lack of standard clinical protocols for the type, dose, or duration for P4 therapy [25,33,34,35].

Nearly all EEC patients (>90%) have been found to have a mutation within the PTEN/PI3K/AKT pathway, leading to increased AKT activity [36]. E2 can also activate the AKT signaling pathway [37], enhancing cell proliferation [38]. AKT activation results in decreased transcription of progesterone receptor (PGR) form B (PR-B) in Ishikawa cells as well as *Pgr^cre/+^Pten^f/f^* mouse models of EEC [39]. Communication between endometrial stromal and epithelial cells via P4 and its receptors (PGR) is critical for normal endometrial function [40]. First, endometrial epithelial proliferation is repressed through PGR signaling [40,41]. Second, stromal PGR signaling is important for hormone responsiveness in EEC [42]. Thus, PGR signaling is vital for epithelial–stromal crosstalk. Studies strongly suggest that loss of PGR or P4 signaling pathways [43] and activation of the PI3K/AKT/mTOR pathway [44,45] lead to P4 resistance in various uterine diseases, including CAH and EEC. However, the exact molecular mechanisms that cause imbalanced regulation of the PGR and AKT pathways in P4 resistance and the molecular network involved in P4 resistance are poorly understood.

In CAH and EEC, PGR and P4-regulated genes are downregulated, and the PI3K/AKT/mTOR pathway is activated [44,45], resulting in activated E2 signaling and P4 resistance [46,47]. Mitogen-inducible gene 6 (*MIG-6*; also known as *ERRFI1*, *RALT*, or *GENE 33*) is a 50 kDa adaptor protein [48]. Down-regulated expression of MIG-6 has been observed in human EEC [49,50,51], lung cancer [52], papillary thyroid cancer [53], and breast carcinoma [54,55,56], suggesting that MIG-6 has a human tumor-suppressor role. Decreased MIG-6 expression can result from mutation of the MIG-6 coding region (in lung cancer) [52] or from MIG-6 promoter methylation (in papillary thyroid cancer) [53]. However, in EEC the cause of MIG-6 loss remains elusive.

In this study, we found that MIG-6 levels are lower in human CAH and early-stage EEC compared to the control group. After P4 therapy, the P4-responsive EEC group exhibited a significantly higher MIG-6 expression compared to the P4-resistant EEC group. Our findings suggest that loss of MIG-6 and PGR as well as activation of pAKT led to P4 resistance in CAH and EEC. Furthermore, MIG-6 function is critical for proper P4 responsiveness in the endometrium, and its loss is associated with P4 resistance in P4-resistant CAH and EEC.

## 2. Results

### 2.1. MIG-6 Levels Are Significantly Lower in Human CAH and Early-Stage EEC Compared to Controls

**Figure 1:**
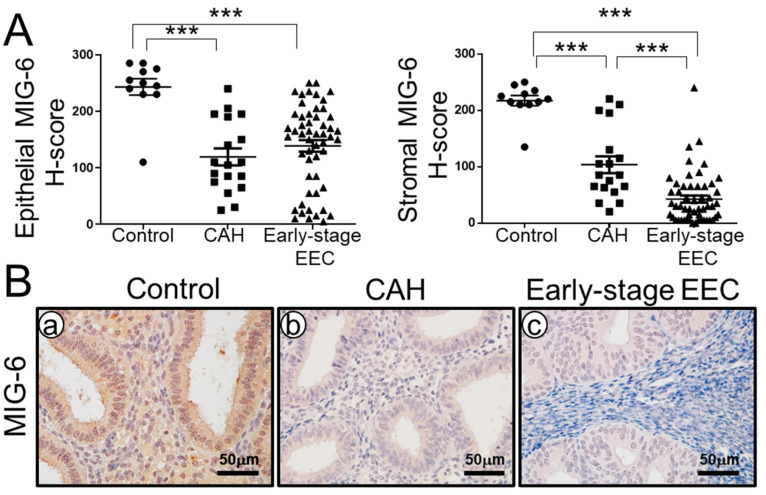
MIG-6 is downregulated in CAH and early-stage EEC. (**A**) H-score of MIG-6 showed that the MIG-6 expression is significantly lower in CAH (n = 18) and early stage (I and II) of EEC (n = 53) compared to control (n = 11). (**B**) Representative MIG-6 expression in control (**a**), CAH (**b**), and Early-Stage EEC (**c**) by immunohistochemistry. *** *p* < 0.001.

To examine the role of MIG-6 in CAH and early-stage (I and II) EEC, we first examined levels of MIG-6 in endometrial biopsies from patients with CAH (n = 18) and early-stage (I and II) EEC (n = 53) and controls (n = 11) using immunohistochemistry (Figure 1). Our results of immunohistochemistry and semi-quantitative analysis revealed that the expression of MIG-6 was detected in the endometrial epithelial and stroma cells from controls. However, MIG-6 expression was significantly decreased in endometrial stroma and epithelium from women with CAH and early-stage (I and II) EEC compared to controls (Figure 1A). H-score analysis revealed that MIG-6 protein expression in the endometrial epithelial cells from women with CAH (119.44 ± 15.04) and EEC (138.87 ± 10.20) was significantly lower compared to women without endometrial cancer (controls, 243.18 ± 14.62, *p* < 0.001). These stromal MIG-6 expressions in CAH and early-stage EEC were lower in stroma cells compared to controls (217.18 ± 9.17, *p* < 0.001). In particular, stromal MIG-6 expression in early-stage EEC (42.64 ± 6.19) was significantly lower than CAH (103.50 ± 14.93, *p* < 0.001). These results suggest that MIG-6 has a tumor-suppressor role in CAH and EEC.

### 2.2. MIG-6 Levels Are Significantly Lower in P4-Resistant EEC Compared to P4-Responsive EEC Following P4 Treatment

**Figure 2:**
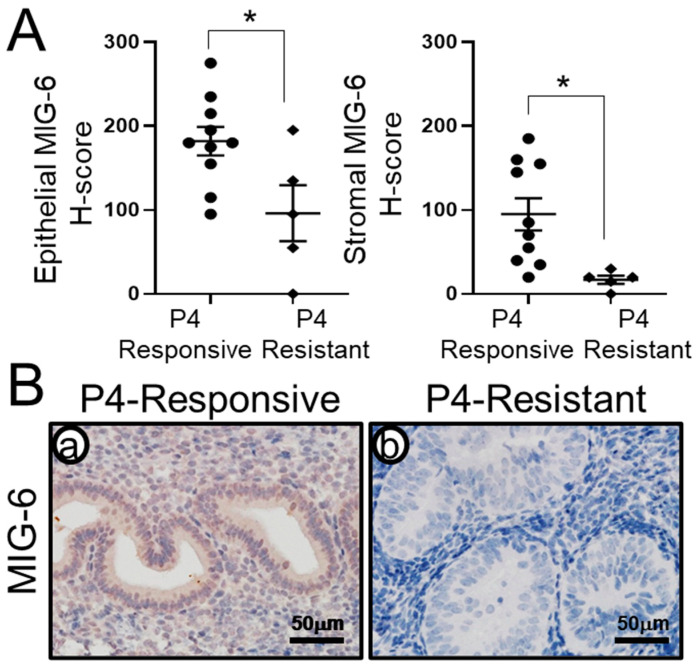
Upregulation of MIG-6 expression is important to overcome P4 resistance in human. (**A**) H-score of MIG-6 showed that MIG-6 expression is significantly higher in P4-responsive women (n = 10) compared to P4-resistant women with CAH or EEC (n = 5) after P4 treatment. (**B**) Representative MIG-6 expression in P4-responsive (**a**) and P4-resistant (**b**) women by immunohistochemistry. * *p* < 0.05.

To determine whether MIG-6 levels are correlated to P4 responsiveness in the human endometrium, we examined the expression of MIG-6 in 15 women with CAH or early-stage (stage I and II) EEC who had received P4 therapy. Histological analysis [57] revealed that 10 out of 15 patients were responsive to P4 treatment (P4-responsive group), whereas 5 out of 15 patients were not responsive to P4 treatment (P4-resistant group). Immunohistochemistry of MIG-6 showed that MIG-6 expression was significantly lower in the P4-resistant group compared to the P4-responsive group (Figure 2). H-score of MIG-6 levels was examined in endometrial stroma and epithelial cells from the P4-responsive and the P4-resistant group for the detail analysis. H-score analysis showed that the P4-responsive group exhibited an increase of MIG-6 expression in epithelial (182.00 ± 16.85) and stromal (95.00 ± 19.15) cells compared to P4-resistant group (96.00 ± 33.33 and 17.00 ± 4.90, respectively, *p* < 0.05). These results suggest that MIG-6 has a critical role in P4 responsiveness, and loss of MIG-6 may cause P4-resistant CAH and EEC.

### 2.3. Recovery of Stromal PGR Levels in P4-Responsive Human CAH/EEC after P4 Treatment

**Figure 3:**
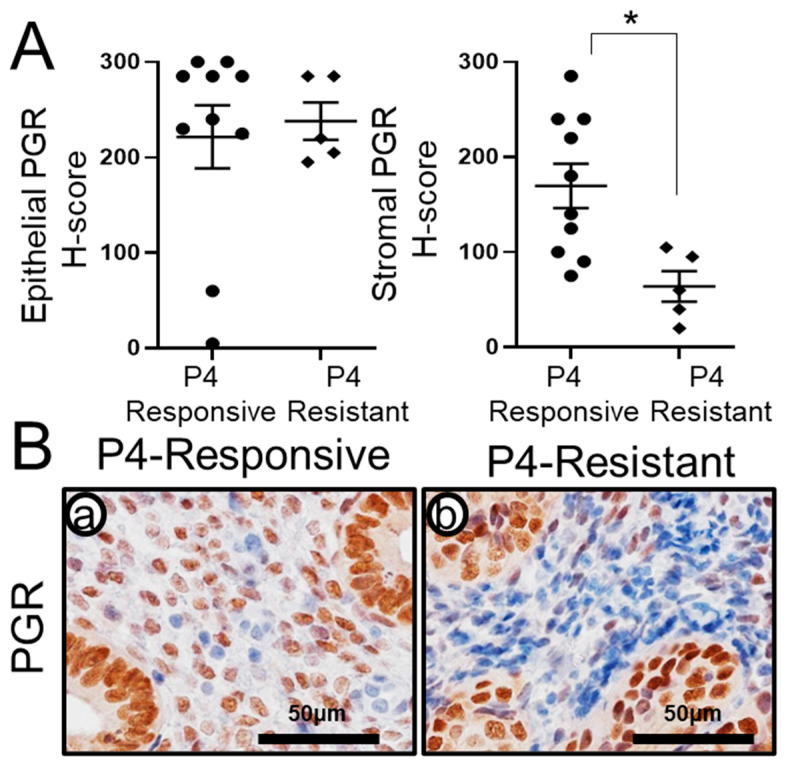
Stromal PGR expression is upregulated in P4-responsive women after P4 treatment. (**A**) H-score of PGR showed that stromal PGR expression is significantly higher in P4-responsive women (n = 10) compared to P4-resistant women with CAH or EEC (n = 5) after P4 treatment. (**B**) Representative PGR expression in P4-responsive (**a**) and P4-resistant (**b**) women by immunohistochemistry. * *p* < 0.05.

One potential molecular cause of P4 resistance is a loss or attenuation of PGR expression [58,59]. Therefore, we examined the levels of PGR in the P4-responsive and P4-resistant groups using immunohistochemistry. As we expected, we found that stromal PGR expression was recovered in the P4-responsive group, whereas loss of PGR expression was found in the P4-resistant group. The quantification analysis of PGR immunohistochemistry using H-score showed that epithelial PGR expression was not different between the P4-responsive group (221.50 ± 32.97) when compared to the P4-resistant group (238.00 ± 19.60). However, stromal PGR levels are significantly lower in the P4-resistant group (64.00 ± 16.08, *p* < 0.05) when compared to the P4-responsive group (169.50 ± 23.30) (Figure 3). Our results suggest that stromal PGR expression contributes to P4 responsiveness in the P4-responsive group.

### 2.4. MIG-6 Levels Have a Reverse Correlation with pAKT in Human CAH/EEC

**Figure 4:**
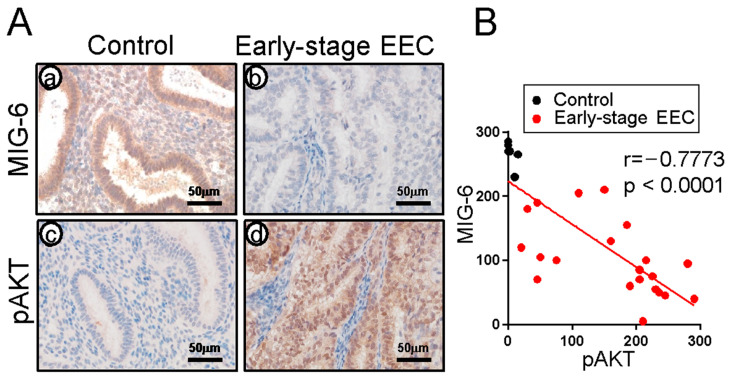
MIG-6 and pAKT expression in early-stage EEC. (**A**) Attenuation of MIG-6 and activation of pAKT were observed in the early stages (I and II) of EEC (**b**,**d**) compared to Control (**a**,**c**) by immunohistochemical analysis (n = 21). (**B**) A reverse correlation MIG-6 and pAKT in early-stage EEC.

As P4 resistance is related to hyperactive AKT signaling in endometrial cancer cells [39,60], we performed immunohistochemistry of MIG-6 and pAKT (phospho-AKT at Ser473) in controls (n = 6) and women with early-stage EEC (n = 21) to examine whether MIG-6 and pAKT expression is correlated in early-stage EEC. Next, we quantified the expression of MIG-6 and pAKT by H-score analysis. Early-stage EEC showed lower expression of MIG-6 (102.14 ± 12.57) and higher expression of pAKT (161.90 ± 18.85) compared to control group (266.67 ± 7.92 and 4.67 ± 2.60, respectively). We found a significant reverse correlation between MIG-6 and pAKT proteins in the early-stage EEC group (Spearman correlation coefficient r = −0.7773, *p* < 0.0001) (Figure 4). These data suggest that MIG-6 mediates P4 signaling as a negative regulator of AKT in human CAH and early-stage EEC.

## 3. Discussion

P4 has been used clinically to treat CAH and EEC in patients wishing to preserve fertility or who have co-morbid conditions preventing definitive surgery [27,28,29]. However, P4 therapy response rates vary, and the molecular mechanisms behind P4 resistance are poorly understood. In this study, we sought to clarify the expression pattern of MIG-6, a P4-responsive gene in the endometrium and examine its relationship to conservative P4 treatment of CAH and EEC. Previous data show MIG-6 expression is higher in the human endometrium of the early secretory phase compared to proliferative phase [50]. During the human menstrual cycle, P4 levels rise at the early secretory phase, which suggests *MIG-6* is a P4-responsive gene in the human endometrium [50,61]. Furthermore, MIG-6 functions as a tumor-suppressor gene in established mouse models of EEC [62]. Down-regulated MIG-6 expression is observed in human CAH and EEC [49,50,51], lung cancer [52], papillary thyroid cancer [53], and breast carcinoma [54,55,56]. Decreased MIG-6 expression can result from mutation of the MIG-6 coding region (in lung cancer) [52] or from MIG-6 promoter methylation (in papillary thyroid cancer) [53], but in CAH and EEC, the cause of MIG-6 loss is unknown. Taken together, these previous findings suggest that MIG-6 may play a role in the response to P4 therapies.

Using IHC, we found that MIG-6 protein expression in the endometrial epithelial and stromal cells from women with CAH and EEC was significantly lower when compared to normal endometrial controls from the mid-secretory phase of the cycle (Figure 1). This result confirms/is consistent with our/the previous report that MIG-6 exhibits reduced expression in EEC and highlights, for the first time, that MIG-6 loss also occurs in CAH, the established precursor for EEC [50]. We also found a more significant decrease of stromal MIG-6 in EEC as compared to CAH (Figure 1).

Next, we examined the expression of MIG-6 in CAH and EEC from women who had undergone conservative non-surgical P4 therapy. We noted increased epithelial and stromal MIG-6 expression in P4-responsive women as compared to P4-resistant women (Figure 2), who demonstrated low MIG-6 levels. Importantly, stromal PGR expression was recovered in the P4-responsvie group, whereas the P4-resistant group exhibited the loss of stroma PGR expression (Figure 3). Studies indicate the stroma surrounding cancer cells are important in tumor development and progression [63]. Interaction between neoplastic cells and the stroma will be a critical factor during tumorigenesis of CAH and early-stage EEC. Endometrial stromal cells have a regulatory role for growth and differentiation of the overlying epithelium [64], demonstrating the stromal cells’ paracrine role in endometrial function. These findings suggest an important role of stromal MIG-6 and PGR in the development and progression of endometrial tumorigenesis.

Although the sample size is small, this is the first report to show a strong relationship between MIG-6 levels and P4 responsiveness in CAH and EEC. Our findings will help in understanding the pathophysiology of P4 resistance in CAH and EEC and improve nonsurgical approaches to P4-resistant CAH and EEC. One limitation of our study is that all our protein expression analyses used immunostaining on human endometrial tissues. Therefore, we do not provide a molecular mechanism on how MIG-6 mediates P4 responsiveness on endometrial cells. Although our correlation analysis was separately performed on endometrial stromal and epithelial cells, we cannot dissect compartment specific roles of MIG-6 and stromal–epithelial interactions on P4 responsiveness. Finally, all the experiments in this study utilized human biopsy samples from translational studies. While it is the first report to show the potential role of MIG-6 on P4 responsiveness of CAH and EEC, the molecular mechanism of MIG-6 on P4 responsiveness needs to be studied using cell lines and/or animal models. Due to the limitation of clinical human data, the relationship of other clinical data with MIG-6 expression was not studied. The expression of MIG-6 associated with other clinical factors, such as menopausal status, age, and BMI, needs to be further studied. Therefore, our findings still need to be validated in human samples.

PGR exists as two isoforms, namely PR-A and PR-B, that are transcribed from two different start sites in the same gene [65]. In vitro studies suggest PR-B is the predominant isoform responsible for P4’s tumor-suppressive action in the endometrium [66,67]. PR-B is a strong transactivator in response to P4, whereas PR-A is less active and, in most cases, inhibits transcriptionally active PR-B [43,68,69,70,71,72,73,74]. MIG-6 may regulate the PGR signaling through protein–protein interactions, as MIG-6 interacts with PR-A but not with PR-B. In addition, alterations in the ratio of PGR isoforms have been observed in the CAH and EEC [75]. Therefore, the determination of how MIG-6 regulates PR-A and PR-B signaling by assaying PGR expression and activities is needed in further studies. Furthermore, understanding molecular differences between stromal and epithelial cells will be critical to identify the alternative molecular targets associated with P4-resistance in EEC.

Cancer Genome Atlas data show that over 90% of EEC patients have a genetic aberration in the PTEN/PI3K/AKT pathway, leading to increased AKT activity [36]. P4 resistance was reversed by inhibition of PTEN/PI3K/AKT signaling through a PGR-dependent, non-genomic, rapid signaling mechanism in human EEC cells [44]. AKT reduces PGR protein expression levels in breast cancer cells, EEC cells, and endometriotic stromal cells [76,77,78]. Inhibition of AKT in conjunction with P4 (R5020) treatment upregulates a subset of PR-B target genes in Ishikawa cells [39]. We observed the reverse correlation between MIG-6 and pAKT in early-stage EEC (Figure 4). While downstream mechanisms of AKT/mTOR activation are well-known in EEC [79], negative regulation of AKT activation via other pathways in EEC is poorly understood. Therefore, understanding of the role of MIG-6 as a critical negative regulator of AKT in human CAH and EEC will be important to open a new avenue of research to unravel CAH and EEC mechanisms and P4 resistance in CAH and EEC.

MIG-6 is a P4 target gene in the human endometrium [50]. P4 effects are mediated by PGR expression [80]. A potential molecular cause of P4 resistance is loss or alteration of PGR expression [58,59]. Stromal PGR expression correlated with favorable response to progestin treatment in women with CAH and EEC [81]. Endometrial stromal cells have a regulatory role in growth and differentiation of the overlying epithelium [64], demonstrating the stromal cells’ paracrine role in endometrial function. P4 signaling disruption unleashes unopposed estrogen (E2) stimulation, which causes CAH to develop into EEC [20,21]. Our study showed that P4-responsive women with CAH and early-stage EEC had higher stromal PGR and MIG-6 expression than P4-resistant women. Our results support that stromal MIG-6 expression is critical to P4 responsiveness, and its loss results in P4 resistance in humans with CAH and EEC. In addition, the AKT/mTOR signaling pathway is hyperactivated in human CAH and EEC [82,83,84,85,86], and P4 resistance is related to hyperactive AKT signaling in EC cells [39,60]. We found a significant inverse correlation between human MIG-6 and pAKT proteins in the early-stage EEC group. These data suggest that MIG-6 negatively regulates AKT phosphorylation in CAH and EEC development.

The mechanism for MIG-6 as a significant modulator in the regulation of PGR and P4 signaling to P4 responsiveness in CAH and EEC is required in the following study. In addition, negative regulation of AKT and the relationship between AKT and P4 signaling have not been studied in CAH and EEC. It also needs further study.

Our results indicate that stromal MIG-6 is critical for proper P4 responsiveness and that its loss results in P4 resistance in human CAH and EEC. This work will help unravel how MIG-6 is involved in P4 action during tumorigenesis and may open a new path to therapy for P4-resistant CAH and EEC while helping women maintain endometrial functions.

## 4. Materials and Methods

### 4.1. Human Sample

In total, 11 controls, 18 endometrial hyperplasia, and 53 early-stage (I and II) endometrial cancer samples were used for this study. All samples were de-identified and obtained as formalin fixed paraffin-embedded sections and their use approved following Institutional Review Protocols. Normal human endometrial control samples from 11 women without endometrial cancer were collected from the secretory phases at Wake Forest Baptist Health. Samples of patients with CAH and EEC post synthetic progesterone treatment (n = 15) were obtained from the Spectrum Health Universal Biorepository. Early-stage (I and II) endometrial cancer samples were obtained from The University of Texas MD Anderson Cancer Center (n = 13) and Spectrum Health Hospital (n = 40). We purchased a human paraffin-embedded tissue microarray slide that contained 18 endometrial hyperplasia samples from U.S. Biomax (Cat# UT240. Rockville, MD, USA).

### 4.2. Immunohistochemistry

Immunohistochemistry analyses were performed as previously reported [41]. Briefly, uterine sections were exposed to anti-MIG-6 (1:200 dilution in PBS contained 10% normal goat serum (S-1000; Vector Laboratories, Burlingame, CA, USA), Customized antibody by Dr. Jeong Lab), anti-PGR (1:1000 dilution in PBS contained 10% normal goat serum, SAB5500165; Sigma Aldrich, St. Louis, MO, USA), and phospho-Akt (Ser473) (1:500 dilution in PBS contained 10% normal goat serum, CS-4060;Cell signaling, Danvers, MA, USA). The sections were then exposed to anti-rabbit secondary antibody (1:1000 dilution in PBS contained 10% normal goat serum, BA-1000; Vector Laboratories, Burlingame, CA, USA) for PGR and phospho-Akt and anti-mouse secondary antibody (1:500 dilution in PBS contained 10% normal goat serum, BA-9200; Vector Laboratories, Burlingame, CA, USA) for MIG-6 and then incubated in horseradish peroxidase (1:500 dilution in PBS, 43-4323;ThermoFisher Scientific, Waltham, MA, USA). The signal was detected by the Vectastain Elite DAB kit (SK-4100;Vector Laboratories, Burlingame, CA, USA). For immunohistochemistry staining comparison, a semiquantitative grade (H-score) [47] was measured by adding the percentage of intensively stained cytoplasm (MIG-6) or nuclei (PGR) (3×), the percentage of moderately stained cytoplasm or nuclei (2×), and the percentage of weakly stained cytoplasm or nuclei (1×) in representative fields of approximately 150 epithelial cells and 150 stromal cells from 11 controls, 18 endometrial hyperplasia, 53 early-stage (I and II) endometrial cancer tissue regions, and 15 post synthetic P4 treatment; the score range is 0 to 300.

### 4.3. Statistical Analysis

To assess statistical significance of parametric data, we used one-way ANOVA analysis, Tukey’s post hoc multiple range test for three groups, or Student’s *t*-tests for two groups. Spearman correlation coefficient was used to assess correlation. Statistical analyses were performed using GraphPad Prism 9 (San Diego, CA, USA). *p* < 0.05 was considered statistically significant.

## Data Availability

The datasets used and/or analyzed during the current study are available from the corresponding author on reasonable request.
